# Accident and pollution risk assessment for hazardous cargo in a port environment

**DOI:** 10.1371/journal.pone.0252732

**Published:** 2021-06-04

**Authors:** Rafi Ullah Khan, Jingbo Yin, Faluk Shair Mustafa

**Affiliations:** Department of International Shipping, School of Naval Architecture, Ocean and Civil Engineering, Shanghai Jiao Tong University, Shanghai, China; Al Mansour University College-Baghdad-Iraq, IRAQ

## Abstract

The catastrophic environmental, life and monetary losses concomitant to the hazardous cargo accidents have remained a matter of critical concern for the maritime transportation officials. The factors that instigate these accidents while dealing with hazardous cargo in a port environment requires rigorous analysis and evaluation, which still remains in its infancy. In accord to these prevailing issues, this study focusses on the assessment of multifactor risks associated with the dealing of hazardous cargos inside a port. The methodology adopted is the amalgamation of expert judgment and literature review for the identification of factors and developing their causal relationship, while Bayesian Network (BN) for the inference, which was based on 348 past accident reports from the year 1990 to 2018. The results indicate that under normal circumstances, the probability of an accident with considerable consequences is 59.8, where human and management were found to be the highest contributing factors. Setting evidence at the environment and pollution accident to occur, the incidence probability of the “management” is raised by 7.06%. A sensitivity analysis was conducted to determine the most critical factors for the hazardous cargo accident. This study reveals that in order to evade the hazardous cargo accidents and curtail severity of the consequences, the port authorities, concerned government departments and other related institutions should pay specific attention to the qualification, training and attitude of the involved workforce. Moreover, the development and implementation of stringent safety protocols was also revealed to have critical prominence. This study holds practical vitality for enhancing safety and mitigating risks associated to hazardous cargo dealing in a port.

## 1. Introduction

The port industry all over the world has greatly emphasized the establishment of green ports as a leading trend towards the achievement of pollution free environment at ports. This aim has attracted greater consideration and is regarded a common goal to achieve by all countries with maritime transportation [1]. Goods with inflammable, explosive, and toxic properties carry a danger along from one port to another in their transportation [2]. The port industry around the globe thus seriously emphasizes the safer transportation of these hazardous goods. The excessive adaptation of maritime transportation has led to the development of creating greater storage capacities; leading to the increase in throughput of hazardous goods at ports which in turn requires greater consideration towards the safer production and transportation of the concerned hazardous goods [3]. The ports with dangerous goods may face unforeseen accidents leading to greater economic loss and may consequence human casualties.

This development in maritime transportation is not free of the risk associated with the transportation of hazardous goods. This risk on many occasions resulted into dangerous accidents and the most notable accident occurred on August 12, 2015 where fire broke out at the warehouse that was containing dangerous goods at Ruihai International Logistics Co. Ltd. at Tianjin Port, Tianjin Binhai New Area in Tianjin city. The fire led to the explosions and caused serious casualties and economic loss [4]. Another prominent accident in this regard was the Beirut port explosion, where ammonium nitrate was stored and left their for years which eventually resulted in an explosion and brought about serious life, property and environmental losses [5]. Likewise, a serious oil spill of more than 1000 tons from a Japanese ship at Mauritius even after spending millions of dollars on the cleaning processes did serious damage to the local marine environment and conservatories [6]. The significant aspect therefore is to identify key areas which may prevent accidents happenings at ports during the transportation of hazardous material.

About the standard management of ports operations, a study classified certain functional data and provided suggestions that focused the safe operation of hazardous chemical industry at both national and international levels [7]. The safe management of chemical materials was discussed in detail in an information guide provided in this study. This study in detail focused on the areas of necessary measures being adopted in transportation of chemical stuff through various transportation sources like rail, road, air and sea. Various studies have been conducted on maritime and port logistics of chemicals and hazardous cargo which evaluate the risk posed by dangerous goods from various perspectives [8–10]. These studies indicate that human factor is one of the leading accident causation factor. Likewise proper documentation, warehouse management, equipment and technology, natural factors and container handling and packing are acknowledged as critical factors for hazardous cargo safety.

A study suggested a well-established department which should maturely consider the procedural application of an efficient method for the assessment of fires’ risk at ports [11]. Various studies in past have discussed the standard management to minimize the risk of accidents at ports; but the recent growth in sea port operations and maritime transportation indeed requires research to be conducted on every level of operations to minimize the risk of accidents in port operations and transportation. The available studies provide a solid research foundation for researchers to focus the operational classification and facilitate the safety insurance during the transportation of hazardous goods. The accidents at ports symbolize the governance of ports at national level and indicate serious threats of loss during hazardous goods transportation; if not addressed timely [3, 12]. The need of the hour indeed is, to devise an innovative procedural mechanism which should focus to limit the risks of hazardous transportation as its primary concern.

The hazardous cargo risk could be evaluated and assessed through different tools. Some research studies adopted analytical hierarchy process (AHP) theory for the overall analysis [13, 14]. Bayesian networks are acknowledged as a robust tool in the risk assessment domain, and its use in the maritime transportation sector is considered highly reliable, efficient and powerful tool, but considered still in its infancy [15]. Though there are issues associated with BNs as they require a lot of data to set up the prior probabilities, however, they widely employed in the maritime transportation risk assessment domain [16]. The former or prior probability is the central point of dependence in the Bayesian model as it focuses the data obtained from past accidents, related literature and expert judgment for future prediction and analysis. BNs that are based on actual data from past accidents are highly favored for their practicality and accurate results. BNs have been extensively employed in the maritime and hazardous cargo accident risk assessment studies [17–20].

The risk associated with the handling of dangerous goods in a port environment are related to various aspects of human, the port environment, infrastructure and facilities itself and issues with the port authorities and governing bodies. The analysis of these factors holds critical prominence to advocate the right resources towards right issues at a right time. A reliable quantification of the concomitant risk in various scenarios provides a significant support in the decision making associated with safety management and risk mitigation. As discussed earlier, recently there have been many catastrophic hazardous cargo accidents at ports and shores and have been playing havoc to the human life, property and environment. However, there is a serious lack of studies that focus on the hazardous cargo accident risk in the port environment that not only focus on the accident risk, but also the risk posed to the port and surroundings environment.

Therefore, in accord to this serious gap in the literature, this study is focused on the highly desired multifactor risk assessment of hazardous cargos in a port environment. Employing the widespread acceptability of data based BNs, this study aims to develop a BN model. The developed methodology and model provides useful insights into the effective management of hazardous cargo in a port environment in any region and geography in concurrence to the locally prevailing conditions. This study employing the past accidents data, determines the most prominent and decisive factors in developing an efficient, reliable and robust approach for the hazardous cargo handling and developing sustainable safety plans for port authorities, neighboring environments, supply chains and governments. The results of this study holds practical vitality for the process safety professional, decision making bodies, port authorities and governments in enhancing their efficacy in waning the accidents frequency and circumventing their cataclysmic consequences.

The rest of the paper is arranged as introduction being followed by literature review, section 3 provides the adopted methodology, while, section provides the detailed hazardous cargo environment and pollution accident risk assessment, section 5 provides the conclusion of this study and section 6 provides the references incorporated in this study.

## 2. Literature review

The continuous growth in the traffic of freight cargo has emphasized the need of long term sustainability of such growth; playing the role of a key element in devising global policy debate on trade enhancement and environmental protection [21]. Maritime transportation involves industrial and port-vessel activities. The industrial activities at port are comprised of all those activities which concern oil terminals and both chemical and petrochemical plants. While on the other hand, the port-vessel activities are activities which concern the loading and unloading consignment, oil jetties and its searching [22]. Besides, concerning their situation in coastal areas, ports are undoubtedly the most intricate structural systems from environmental protection perspective because a range of goods related activities are performed there [23]. This aspect as a central point of discussion is discussed and recognized accidental spills as the chief reason behind water pollution [24]. Port accidents are described as the most frequent accidents which accumulate 51% of the release that pollutes water. Furthermore, the record port accidents ratio of (59%) is caused and associated with oil spills [23]. Consequently, the rapid spread of pollution due to oil spills is one of the most serious problems faced at ports during consignment loading, bulk liquid, fuel supply and navigation.

Thus, site-specific strategies at regional level are to be defined, so that the port activities may well be facilitated on emergency level. Particular considerations should be given during this procedural implementation to all related oceanographic, meteorological and environmental circumstances to restrict the spread of pollution, including that which is caused by the spills [25]. Consequently, the analysis on oil spills should consider the domains of (i) an exact spatial framework for definite danger evaluation (ii) apposite methodical data which may result into authentic approach towards risk assessment [25–27].

Environmental risk analysis (ERA) gives the estimated calculation as to what level the spills, fires or explosions may cause pollution under certain specific circumstances and geographical location [28]. Furthermore, ERA serves as an essential requirement for the overall process of spatial planning which incorporate the improvement of ‘inventories and maps’ to sustain emergency preparation along the development of a green supervision design; leading to the execution of effective risk management [29, 30]. Presently, ERA for its functionality is not facilitated by any worldly adapted and acknowledged standard method. There is no globally accepted standard method to be applied in ERA. Nevertheless, greater considerations should be given to emphasize the role of ERA towards its approach in handling oil spills with specific illustration to all those tools which define hazardous situations and support the analysis of spatial risk management [31].

Concerning the recognition of hazards, ERA with regard to spills, fires, explosions and leakages their associated scenarios should elaborate its controlling mechanism. The systematic procedure of ERA addresses the identification of danger at first level to classify as how the stressors may get exposed [32]. The identification of environmental dangers has been down through the adaptation of various innovative tools but none exactly defines the nature of accidents to classify its types in the ERA [23]. Data collected through the recorded accidental spills, fires and explosions are utilized for this procedural functionality. The databases in this regard, for instance the FACTS database, are refined with the broader scope to establish international conventions (OPRC) (UN, 1995) however, this primarily reports outsized spills. Though, 95% of the overall spill amount is comprised of the small and medium spill ranges which normally occur at ports and oil terminals during the loading and unloading of consignments with the estimated ratio of 40% to 29% respectively [33, 34]. The overall databases for these small and medium sized accidents are usually maintained as records by the concerned local port and maritime authorities. The further analysis based on the database highlights the actual location and facility which may experience accidents with a detail of the specific causes and consequences [23]. There is this factor of inaccurate database which may not efficiently characterize the true nature of hazards as the scope of information is usually very limited in them; giving consideration to discharge and its appearance. The appearance of the discharge and its source is helpful in identifying pollution.

The proper definition of ERA for the methodical control over oil spills and hazardous cargo accidents is done through the proper meteorological and oceanographic representation of ERA scenarios. In ERA with port-specific location, the variability of (met ocean) tools is not comprised as representative risk component [35]. Various authors in their studies have focused on the met-ocean conditions to examine the exact estimate of affected offshore and coastal area [36]. The basic functionality of these methodological approaches is well defined through its bases in forecasting systems because the nature of their adaptation is purely operational [37]. Nonetheless, the emergency based planning procedures require concentrating on the aspect of prevention. The proper and well defined statistical results in contingency planning are very significant to comprehend the hazardous cargo accident scenarios.

The assessment of risk can never be ignored as the most significant concern in hazardous industrial zone, specifically in maritime transportation. The key concern is to identify all those indicators which adversely affect the safety and thus to minimize every possible risk [38]. The happening probability of an accident and its related adverse consequences are defined as risk. The risk and its consequences in maritime transportation usually adversely affect the economic stability in terms of monetary losses, human harms in terms of casualties and environmental pollution. A Formal Safety Assessment (FSA) was introduced by the International Maritime Organization to manage the functionality of Maritime Transportation System (MTS). The MTS functionality scope is affected by the lack of available historical data. The efficient assessment of risk is made possible by the prior knowledge from experts who have worked in this specific domain, and this knowledge is a significant source of information to base risk assessment on it [39]. One the other hand, the challenge for all models based on prior knowledge is that the data available is reactive instead of proactive facilitation. A research study highlights that the reactive approaches are usually passive and their limited systematic approach does not consider the changes, variables or shortcomings as sources affecting the overall forecast [40, 41]. Certain frameworks in that regard are considered available sources to calculate probabilities and consequences caused by accidents.

The complex system of MTS along with other human, environmental and organizational factors contribute towards the uncertain functionality scope of MTS [42]. Right from its introduction and adaptation, the Quantitative Risk Assessments (QRA) has been attributed great appreciation as compared to qualitative assessments in maritime transportation. The basic approach followed in (QRA) blend expert knowledge and data [43, 44]. A number of QRA models have adopted tools like fault or event-tree analysis (FTA, ETA), evidential reasoning (ER), Bayesian Belief Networks BBNs [16, 45–49]. Researchers in maritime risk assessment have worked on literature review from different perspectives and thus provided wider scope studies to fill the knowledge gape.

Efforts made possible the availability of an outline on QRA models by combining critical research ideas from 87 related academic papers [50]. The major objective in their study was to study risk in collision and grounding through the adaptation of ETA and traffic flow theory. A study discussed the foundation matters; risk analysis in maritime transportation was further analyzed through defining and scientific outlooks [51]. Amid all adapted approaches in maritime risk assessment the BBN approach efficiently classifies the dependencies related to a certain accident through conditional probability tables (CPTs) [52]. In addition to that, BBN has been celebrated for certain other advantages like those of inverse inference ability and network up gradation [16]. Researchers have in detail studied the comparative sketch of advantages and challenges associated with BBN in maritime transportation [53]. The BBN was authenticated by the results of various studies as the most suitable methodology for risk assessment in maritime transportation and its related impending decisions. The BBN was further valued for its positive characteristic of auto up-gradation with available new data. The above mentioned reasons favored BBN adaptation in maritime risk assessment and excessively valued its approach towards maritime safety.

However, the use of the BBN in the port hazardous cargo accident risk is new and still in its infancy. Therefore, it is of phenomenal prominence to evaluate the various causation factors of a hazardous cargo accident in a port environment and provide productive results to aid decision making, devising policies and developing safety management and risk mitigation systems.

## 3. Methodology

Bayesian networks are an amalgamation of the probability and graph theory, and hence recognized as an effective tool for the analysis and assessment of uncertainties and vagueness associated with the causation factors incorporated to the BN model as nodes and states. The data is processed through several tools like artificial intelligence, decision evaluation and the probability and graph theory [49]. It is regarded as one of the main features of BN that it can successfully process the uncertainty associated with all the data incorporated into the developed model. Subject to its profound features, BN proves to be an effective tool in the risk assessment domain, specifically in the maritime transport sector [48]. However, a point of protuberant concern here is that the matter and issue intended to be studied should thoroughly be checked for its consistency with BNs.

The process of development of a BN model encompasses the recognition of the relevant and influencing factors, developing a causal relationship between these factors and demonstrating it through a proper Directed Acyclic Graph (DAG). Another prominent stage in this regard is the data incorporation and quantification. In this study, the identification and selection of the variables and the development of causal relationship among the nodes was done in concurrence to expert judgment and available related literature. While the data for inference and quantification was derived from past accidents occurred during 1990 and 2018. The calculation of probabilities and conditional probability tables (CPTs) was done through parameter estimation. Parameter estimation is an inbuilt feature of the BayesiaLab software package that could be utilized when the related data set has been associated with the developed model. All the fundamental work including the development of model, data association, calculation of the probabilities and CPTs, inference and the sensitivity analysis was conducted in the academic version of BAyesiaLab 8 software, which is a comprehensive, consistent, efficient and robust tool in BN domain. The adopted methodology has been elaborated in detail below.

### 3.1. Development of nodes and causal relationship

To develop a BN model, the initial stage is to identify the select the variables that affects the scenario under study. After the variables have been selected, these are represented as nodes and states in the BN environment. The next stage is to identify and develop the causal relationship between these nodes and impart it an appropriate graphical representation, termed as the direct acyclic graph. The development of DAG holds critical prominence in imparting a justified development and interpretation of the cause-consequence relationship. However, to construct these relationships through the incorporation of mathematical expressions is renowned as a task next to impossible. A DAG consist of nodes and arcs. The node from which the arc arises is called as the parent node and it ends at the child node.

The most important and significant aspect in the BN domain is the availability of data. It could be achieved from the relevant literature, accident reports and the databases and the concerned authorities. Another prominent aspect in this regard is the expert judgment, which can verily be employed as data. However, to augment the reliability and pragmatism, and fade the ambiguity, the expert judgment should be replaced by the real data whenever becomes available. Almost all the governments and various international organizations keeps a record of the accidents that have occurred in their jurisdiction fulfill their scrutiny criteria. Hence, whenever an accident takes places, it is investigated thoroughly to its details to determine the nature of the accident, identify the factors that caused the accident along its sequence of occurrence and quantify the severity of consequences. Reports containing such information can serve as a potentially rich data source for the Bayesian model.

However, such reports cannot be selected and used on random bases. A strict and comprehensive selection criterion is developed for it in concurrence to the expert judgment and literature review that identifies and shortlists the variables and nodes to be considered for the proposed study. Hence, only those reports are considered for the study which satisfies the developed scrutiny and imparts required data on all the selected variables. In this study, the selection of nodes, states, their causal relationship and the criterion for reports selection was done in concurrence to the available literature in this domain. In concurrence to this criterion, the DAG was developed, data file was developed and arranged in a format that was in compliance with the BayesiaLab conditions. The selection of nodes, development of causal relationship and the model was also discussed with the subject matter experts working both in the academic and research fields and the maritime and port industry. Hence, the final form of the model went through a process of deliberations and changes, unless it was agreed upon by all the participants.

### 3.2. Calculating probabilities and CPTs

Once the identification and selection of the nodes is completed, their causal relation has been identified and the model is developed, the next stage is associated with probabilities calculation. The quantification of probabilities and CPTs could be achieved through expert judgment, the retrieved accident reports or an amalgamation of these two. The quantification can be achieved using various tools like Bayes theorem, logistics regression and maximum likelihood estimation. In situations where the real data is available and can be associated with the BN model, the probabilities and CPTs can be calculated through the “Parameter estimation”. Parameter estimation is an inbuilt feature of the BayesiaLab software package and works on the principle of maximum likelihood estimation. The mathematical expressions and further details of the maximum likelihood estimation have been provided in an earlier study of the authors and can be consulted from there [49].

### 3.3. Sensitivity analysis

Sensitivity analysis is conducting to determine the most critical factors for a specific result of a specific scenario in a BN model. Sensitivity analysis imparts the magnitude or strength of the two way association between the parent and child node. One of the prominent aspect to be considered while conducting sensitivity analysis is the selection of number of parameters. The concept of conducting the sensitivity analysis is making variations in the parameters and analyzing its impact on the other nodes or parameters. It could either be simple in which variations are made in only one parameter, or complex in which multiple parameters in a CPT are considered. The reliability is believed to increase with increase in number of parameters and complexity. Though it requires a comprehensive and far-reaching understanding of the joint probability distribution and network parameters, but the sensitivity analysis involving multiple parameters from multiple CPTs is believed to be the most authentic, reliable and holistic [48, 49]. BNs are believed to exhibit a robust and practical interaction between the considered variables through the induced variations in the selected parameters.

In this study, the sensitivity analysis will be conducted through an inbuilt feature of the BayesiaLab software package called “Tornado Charts”. These tornado charts displays the maximum and minimum contribution of all the variables in a model towards a specific node and state which is specified as the target node and state. The values of these charts could also be converted in to numeric values and presented in tabular form. Apart from the sensitivity analysis, in order to determine the consistency and level of confidence in the produced results, a BN model has to verify the following conditions acknowledged as the validation of model [2, 5, 51, 52]. These conditions could be summarized as, a variation brought in the prior probabilities of parent nodes shall produce a relevant variation in the posterior probabilities of the child node. Similarly, the magnitude of the effect induced by changing the probabilities shall remain greater for the set evidence in comparison to the other sub factors in the model.

## 4. Hazardous cargo risk assessment in a port environment

Though the world has seen many advances and improvements in the port safety system, however, the port environment yet sees the accidents involving hazardous cargo. No port in the world could be considered totally immune to these accidents. These hazardous cargo accidents could occur in any part and activity level inside a port. The past accident reports reveals that such accidents have occurred during the loading and unloading of the hazardous cargo and transporting these cargos in and out of the port. Moreover, such accidents have also been found to have taken place inside the port temporary storage areas. These accidents could result in intermittent fires, catastrophic oil spills and chemical discharges, contamination of the air, earth and water, corrosiveness, explosions and pollution of the surrounding environment. In addition to these environmental issues, damage of goods, properties, port infrastructure and portfolio are also of critical concern. Such accidents could incur huge financial losses.

### 4.1. Specifying nodes and states of BN

Likewise the manifold consequences of the hazardous cargo accidents, the factors which initiate and cause such accidents are also diverse in nature. The nature of these causation factors could either be human, organizational, management, facilities and natural. The human factor is further classified into technical qualification, experience and attitude of the employees involved in the handling of dangerous goods [10]. If the employees lack the necessary qualification required to handle such dangerous goods, it could pose a serious risk. Similarly, a prior experience of handling the dangerous goods also contributes to enhanced safety and any shortcoming or lack of experience on the part of involved employees could initiate a significant threat [8, 10]. Moreover, the attitude of the involved employees also plays a very prominent role in the safe operations associated with hazardous cargo. This attitude could be nature and carelessness of employees themselves, their lack of training, excessive working hours, lack of interest due to improper facilities and pay grade and understanding of the situation [8, 9].

The other most prominent dimension is the organization itself. It stands for the proper warehousing of the dangerous goods at the port environment in concurrence to the rules defined by national and international standard operating procedures [8, 9]. Similarly, the development of their own site, project and work nature based safety regulations also holds critical prominence. Also, the development of a specific department, workforce and organization to deal with the operations, storage and handling of these goods also plays a noteworthy role in evading accidents [8].

Subject to the 348 past accident reports from 1990 till 2018 considered for this study, literature review [42, 54–66] and expert judgment, this study has focused at the important causation factors of the dangerous cargo accidents in a port environment. These factors have been arranged into a set of variables, and hence nodes in the model developed. All the factors considered have been depicted in the Table 1.

**Table 1:** Depiction of the variables considered for this study.

Variable	State ‘0’	State ‘1’	Variable	State ‘0’	State ‘1’
Qualification	Yes	No	Infrastructure	Good	Poor
Attitude	Good	Bad	Equipment	Good	Poor
Experience	Yes	No	Facilities	Good	Poor
Human Factor	No Effect	Influence	Registration	Good	Poor
Warehousing	Good	Poor	Safety Protocols	Good	Poor
Operations	Good	Poor	Management	Normal	Bad
Emergency	Good	Poor	Natural Factors	Normal	Bad
Organizational Factors	No Effect	Influence	Environment and Pollution Risk	Normal	Bad

In the above table, all of the variables are defined as binary in nature and two states as “0” and “1” have been specified for it elaborating its own meaning. Majority of the variables considered are self-explanatory. However, to avoid any ambiguity, the variables under consideration are being clarified.

#### 4.1.1. Human factor

This variable is included to define the role played by human factor in the accident instigation. As discussed earlier, human factor is a prominent accident instigator and in this study three aspects of this variable are considered.

*4*.*1*.*1*.*1*. *Experience*. This sub-factor represent the experience level of the ship crew. It depicts as if the crew was experienced enough while conducting their job or was indicated otherwise in the accident report.

*4*.*1*.*1*.*2*. *Attitude*. This aspect of the human factor depicts the involvement and attitude of the crew or involved personnel towards their job. It encompass the description in accident reports about the job seriousness, follow of rules and commands, abuse of authority, professionalism and the use of alcohol or any other drugs.

*4*.*1*.*1*.*3*. *Qualification*. This sub-factor indicates if the involved staff was qualified enough to do their job.

#### 4.1.2. Organizational factors

Organizational factors are also considered to be prominent accident instigators. It is also a broader domain and hence its different aspects have been considered as sub-factors.

*Warehousing*. The “Warehousing” node stands for the effective storage and management of dangerous goods in the port warehouses.

*4*.*1*.*2*.*1*. *Operations*. The “Operations” node stands for if the port authorities have defined and developed a specific team and organization for the storage and handling of the dangerous goods and also that they are performing their duties in concurrence to the defined regulations.

*4*.*1*.*2*.*2*. *Emergency*. The “Emergency” node depicts the availability and capability of a specific emergency response team that could timely arrive and manage the situation at site in case of dangerous cargo accident.

#### 4.1.3. Facilities

This factor indicates how the accident reports have mentioned the role of involved facilities in accident causation. There could be lack of facilities and their malfunctioning or poor state.

*4*.*1*.*3*.*1*. *Infrastructure*. The “infrastructure” node depicts if the port infrastructure meets the requirements of the dangerous goods handling. It could be good if it meets the standards and there is no objection or bad otherwise.

*4*.*1*.*3*.*2*. *Equipment*. The “Equipment” node demonstrates if the port authorities have all the required equipment and machineries in enough numbers, condition and maintenance backup to ensure the smooth handling and operations of hazardous cargos.

#### 4.1.4. Management

An effective management and conduction of the port activities, standard operating procedures and assortment have a critical role in safe hazardous cargo port operations, which has been depicted by this variable. The different aspects of this variable are given below.

*4*.*1*.*4*.*1*. *Registration*. This sub-factor depicts if all the dangerous cargo have been efficiently registered and the corresponding data have been put on accessible records. This variable indicates if the accident reports have included the entailment of poor or lack of registration of dangerous goods towards accident causation

*4*.*1*.*4*.*2*. *Safety protocols*. The node “Safety Protocols” refers to the development and implementation of effective and up-to-date safety procedures and guidelines. It also stands for the effective supervision and management of the health and safety situation associated with the hazardous cargo.

#### 4.1.5. Natural factors

This variable indicates the role played by natural phenomenon in accident causation. It encompass the effects of rains, storms, winds, thunderstorms, lightening and tsunamis etc.

#### 4.1.6. Environment and pollution risk

The environmental and pollution risk has been classified into two states. Where, the “Normal” state stands for small leakages, insignificant smaller accidents and scenarios in which failure in one or more than other contributory factors had occurred, but the hazardous cargo accident didn’t take place. While, the “Bad” state stands for accidents which had noticeable consequences and pollution from the leakages, fires and explosions.

**Fig 1:**
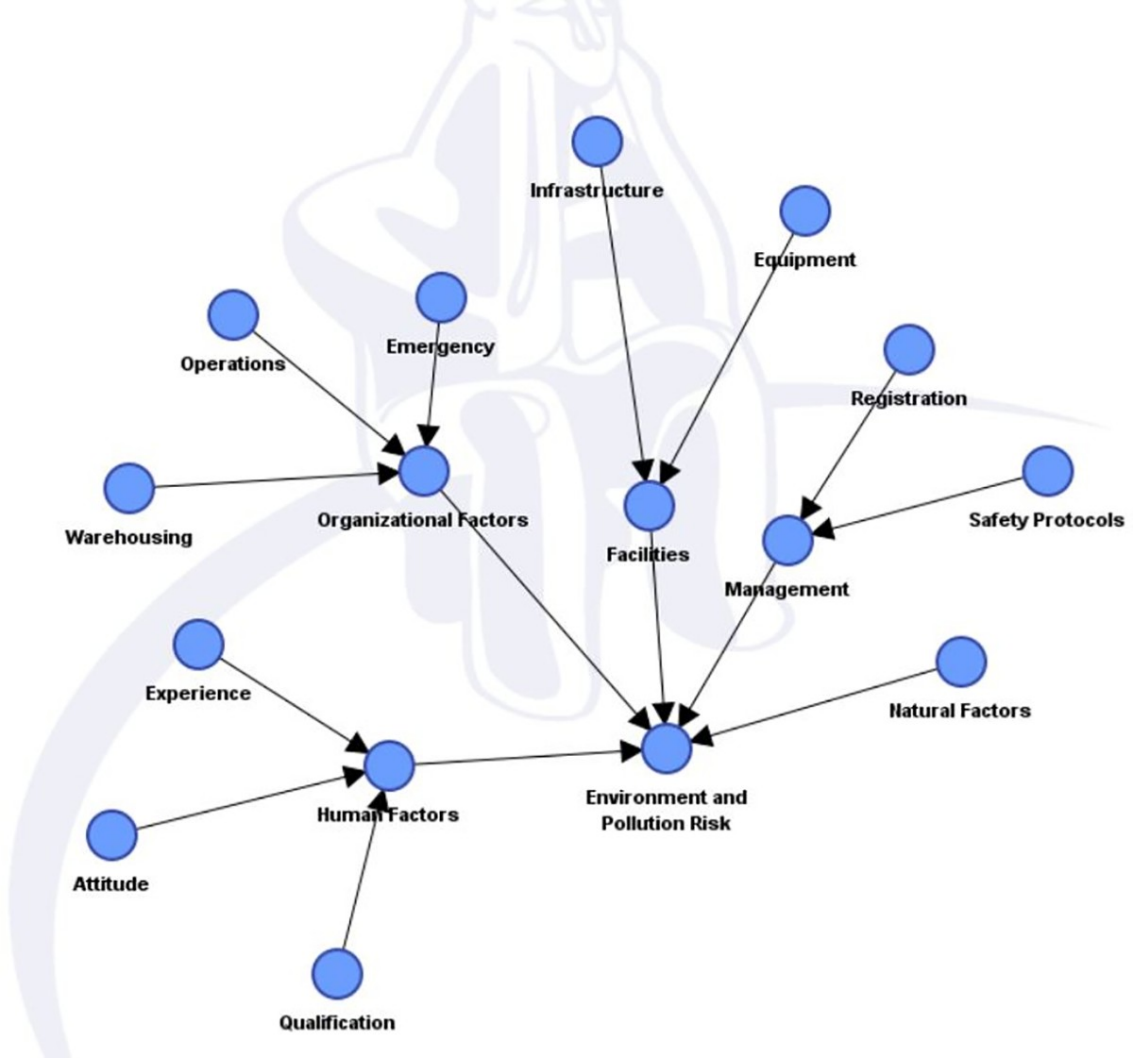
DAG of the developed model.

Once the nodes and states were specified and the data from accident reports was arranged in concurrence to theses states and nodes, the selected nodes were developed into Bayesian Model. This Bayesian Model is also termed as the Directed Acyclic Graph (DAG), where all the connections between the nodes were developed in agreement to the available literature and expert judgment. The model developed for this study is depicted in Fig 1.

All the reports from the duration under study which were satisfying the basic criteria of fulfilling the data availability requirement for all the nodes were set into a data file. This data was then associated with the developed model using the “associate data” feature of the BayesiaLab software. Once the data file was associated, all the probabilities and conditional probability tables were calculated through the parameter estimation function of the software.

### 4.2. Results and discussion

**Fig 2:**
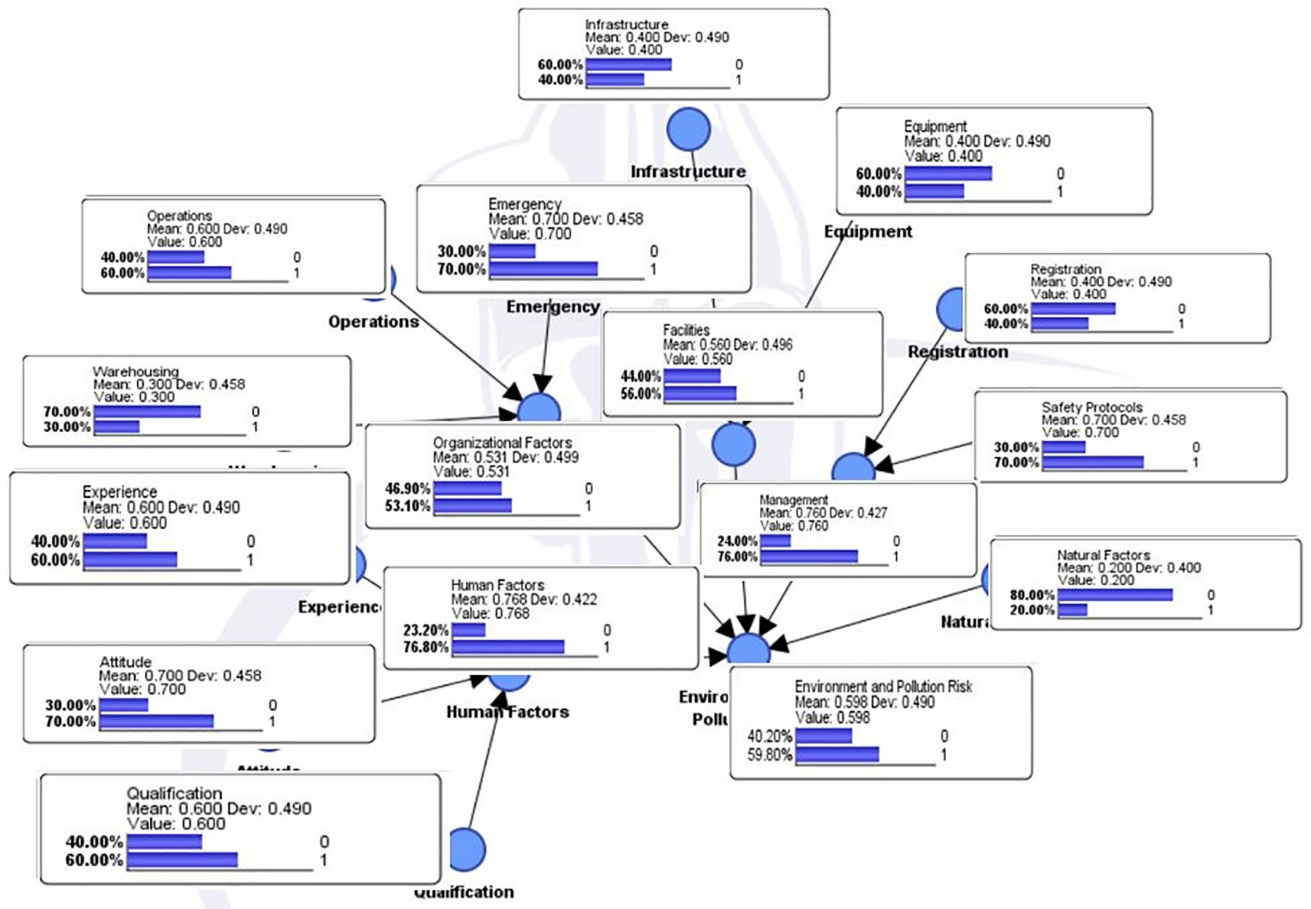
Inference results under normal conditions.

After running the model in the BayesiaLab environment, the inference results drawn as shown in Fig 2, indicates that accidents with noticeable pollution, property and monetary losses had highest occurrence probability of 59.80, while the accidents with insignificant or very minor consequences had a 20% lower rate of occurrence.

Looking at the causation factors in Fig 2, it could be seen that human factor had the highest contribution with a probability of 76.80, which is very prominent and in concurrence to the human factor involvement in the maritime transportation accidents domain. Among the sub factors in Fig 2, the attitude of the staff associated with the dealing of hazardous cargo mattered the most. The human attitude had the highest probability of 70. The attitude of the staff holds critical significance towards the control of hazardous cargo accidents. The staff needs to be evaluated for their vigilance, interests, contribution and enthusiasm towards their duty. A proper policy needs to be devised for the fatigue surveillance and no staff member shall be put under duress for extra working hours or situations which are considered unfavorable for working [10, 67]. Also, the pay grade, safety gear and all other facilities of the staff shall be given due attention as it contributes towards the mind presence of staff in the work and can be very efficient in situations of an undesired event [8]. Moreover, in case of an undesired event or emergency, the situation handling and management is the responsibility of every person within the port area to contain the damage and pollution. This responsibility doesn’t limit to the port personnel only, but extends to the people in the vicinity of the port.

The qualification and experience of the staff both has a probability of 60 in the results under normal situations as depicted in Fig 2. These results highlights the need for the risk mitigation qualification and training on the emergency response measures [8]. The port authorities and the involved department of the government shall not believe that such qualifications and skillset comes on its own, rather they should focus on developing training programs to enhance the skillset of the personnel who has to deal with the hazardous cargos in the port environment. The modern day seaports requires to be highly efficient and safe in order to ensure sustainable businesses and operations. Hence, the port authorities shall focus on the proficiencies of the involved personnel to enable them of effectively tackling various unforeseen situations and handling the undesired events [10].

At the international level, the workers organizations have experienced the need for enhanced skillset required in the modern day seaports subject to the persistently changing job profiles. Therefore, the personnel should be trained and analyzed for their competency on performing their duties related to the management of hazardous cargos. The international maritime organization also enforces that the personnel who deals with the hazardous cargo shall specifically trained for it. This specified training will enhance their risk mitigation capabilities and application along adaptation of the defined emergency protocols [8]. Though how critical this is, port authorities around the globe have been found to violate these recommendations and rules as evident from the accident reports.

In terms of the causation probability, in Fig 2, the second most prominent factor is the port hazardous cargo management capability. It encompasses the prominence of both proper registration and documentation of the hazardous goods, and the development along implementation of the associated safety protocols, rules, measures and standard operating procedures. Lack of the design and implementation or not in concurrence to the cotemporary standards of the related safety protocols, is the highest contributing sub factor in this domain. It also accounts for the lack of or substandard supervision and management of the staff doing their duties and the lack of required latest technology for the hazardous cargo operations [68].

The second highest contributing causation factor in this domain is the proper registration and documentation of the hazardous cargos. The proper and correct documentation holds critical significance for the launch and success of rescue missions in case of emergencies. It could be well elaborated by example that the fire extinguishers required and the safety procedures for the annihilation of a fire initiated by gasoline are much different from that of a fire which is started by kerosene oil, even though both of these materials falls under the same category “flammable liquids” of the international maritime organization. Therefore, accurate documentation is considered to enhance the efficacy of the port safety system and transportation of the hazardous cargos [68].

The third highest contributing causation factor in Fig 2 is the facilities. It encompasses the prominence of the port hazardous cargo related infrastructure and the equipment. Port infrastructure holds critical value as it is an indications of its capability to successfully and reliably accommodate the hazardous cargo operations. Similarly, the availability of the latest technology, modern equipment in the required number, and reliable maintenance along backup is very critical for the port hazardous cargo operations [8].

Now, employing the inverse propagation property of the Bayesian network, evidence is set at the environment and pollution related hazardous cargo accident to occur. It determines the contribution of all the considered factors in a scenario as if the accident has taken place. The highest involvement in this scenario is of the management factor, which experience an increase of 7.06% in its causation probability. While, the highest contributing sub factor in this regard is the safety protocols. It determines the prominence of effective and cutting edge safety protocols and emergency procedures to be in place and their management and implementation be strictly monitored. While, the second most important factor in this scenario is the human aspect. The qualification, experience and attitude of the staff dealing with hazardous cargo has a critical role in the occurrence of a hazardous cargo accident in a port environment. The causation probabilities of all the factors in this scenario are presented in Table 2.

**Table 2:** Probabilities of the causation factors when evidence set at hazardous cargo environment and pollution accident to occur.

Node	State’s Probabilities		Node	State’s Probabilities	
	0	1		0	1
Natural Factors	78.44	21.56	Operations	40.35	59.65
Safety Protocols	23.49	76.51	Warehousing	70.21	29.79
Registration	58.61	41.39	Organizational Factors	47.57	52.43
Management	16.94	83.06	Experience	39.46	60.54
Equipment	63.58	36.42	Attitude	29.73	70.27
Infrastructure	61.95	38.05	Qualification	39.46	60.54
Facilities	49.01	50.99	Human Factors	22.26	77.74
Emergency	30.36	69.64			

Ensuring the hazardous cargo related safety in a port environment is a multifactor based scenario, that specifically revolves around the human, management and organization based factors [10]. The human factor in this regard is considered most critical as it is embedded in the other systems too and plays its role in accident causation and evading in both the poor and efficiently designed systems. From the perspective of warehouse management, not every storage is appropriate for hazardous cargo storage as it requires additional safety and storage measures. Enhanced fire, explosion and leakage control systems are required in such storage units. Also, hazardous cargo cannot be stored next to flammable goods and materials that may aid the fire instigation. Therefore, the port authorities shall assign properly featured areas that has specific capability for the storage of the hazardous cargos eliminating any threat or risk of an accident and hence an environmental catastrophe. In these specifically allocated areas, a strict and efficient supervision requires to be instigated. Port authorities shall devise a warehouse safety management system that is specifically designed for the hazardous cargos and can efficiently handle the storage and transportation for all the categories and stake holders involved [8, 9]. Moreover, the port hazardous cargo safety management system shall be based on the accountability. Where, the employees will focus on their responsibility, skillset and cognizance that would in turn optimize the safety system bearing fruitful results [10].

The port safety system and goods registration protocols shall be aligned with the international and government defined rules [68]. Moreover, these hazardous cargo rules & regulations on management shall be accustomed to the local prevailing conditions in the port environment. All the specific characteristic of the port features shall be amalgamated into the extensive and purpose oriented safety systems of goods transport, storage, and handling and emergency circumstances. Moreover, the registration data and record shall be maintained in compliance to the international standards in a comprehensive way such that it could be readily available for the scientific analysis and research [68]. Moreover, the use of information technology shall be incorporated in the safety management system of the hazardous cargos so that a comprehensive risk management system could be developed not only at the government level but at the international level focusing at the ports with same natural, infrastructure and management features. These technological system shall be developed on the basis of the real time data incorporation so that all the relevant data could be embedded into the system and made readily available. This will enable the system of all the pertinent risks and initiate early warning systems in case of any potential risk or operating system discrepancy.

Another prominent perspective in the port hazardous cargo system safety is the efficiency and appropriateness of port’s relevant equipment and infrastructure [8]. From the infrastructure and equipment perspective, the type, number and maintenance are the key aspects that the port authorities shall pay specific attention to. Moreover, the infrastructure and equipment should be specifically designed and purpose built in concurrence to the geographic, climatic and terminal features. Moreover, in concurrence to the local prevailing conditions, a robust safety and emergency response system should be embedded into the system that will impart the capability of timely and effective measures in case of an accident. The most prominent safety and risk mitigation systems like pollution containment, sophisticated fire alarm and firefighting system should be implanted. Moreover, these systems and equipment must remain installed in enough quantities that enables the capability to mitigate any undesired even efficiently [8].

Additionally, the other most protuberant aspect in this regard is the maintenance of such infrastructure, equipment and safety systems. The port authorities shall conduct regular trainings and workshops on the proper, efficient and safe use of the infrastructure and equipment systems. The concerned staff should be trained on the specific systems uses and must be able to identify the right maintenance and repair at the right time to avoid any unforeseen catastrophic event. Also, the port authorities shall pay keen attention to the international certifications for their equipment and personnel, regular and timely updates of their systems and arrange consistent and systematic calibration of all the in-use equipment and technology.

Inability on the part of personnel, management and authorities and unavailability of the proper infrastructure and equipment can result in serious and calamitous hazardous cargo accidents. These accidents are believed to have multidimensional consequences. It kills, induce injuries, destroys properties, incur huge monetary losses and most importantly, it contaminates and pollutes the natural environment, which in itself is a multidimensional calamity being detrimental not only to the humans, but all the living creatures.

### 4.3. Sensitivity analysis

The purpose of conducting sensitivity analysis is to recognize the most perilous and critical factors or parameters in the developed model and the scenario under analysis. These highly critical parameters are associated with high probability of incidence and involvement in comparison to the other factors considered in the model and study. However, there is no thumb rule or a specified amount of disparity that would turn a variable considerable. Sensitivity analysis is believed to play a significant role in the determination of the most critical factors and variables that if prioritized can considerably evade the happening of an accident and substantially curtail the severity of consequences. However, the ranking classification and selection is highly dependent on the decision maker rather than forecaster or analyst who conducted the analysis.

The environment and pollution risk was set as the target node and state in constructed BN model to conduct the sensitivity analysis. In the BayesiaLab environment, the sensitivity analysis could be determined through the use of “tornado charts” feature. These tornado charts displays the maximum and minimum occurrence and contribution probability of each considered node and variable and hence its effect on the occurrence of the set target node and state. In this study, the minimum and maximum incidence probability values of each variable for the set environment and pollution risk node are incorporated in to a table and presented below in Table 3. The determined critical factors and their difference in probability were also verified by setting evidence at each variable in concurrence to the available literature [49].

**Table 3:** Sensitivity analysis for the environment and pollution accident risk.

Node	Probabilities		Node	Probabilities	
	Min	Max		Min	Max
Management	42.20	65.36	Infrastructure	56.88	61.75
Safety Protocols	46.83	65.36	Natural Factors	58.64	64.48
Facilities	54.45	66.61	Registration	58.41	61.89
Equipment	54.45	63.37	Human Factors	57.38	60.54

The results reveal that the management as overall and the devising and implementation of safety protocols in particular are the most critical factors when it comes to the occurrence of a hazardous cargo accident in a port environment. These factors needs to be given specific attention allocated specific resources for in order to circumvent the occurrence of such accidents. Apart from that, the port facilities in terms of the infrastructure and equipment are ranked as the second most critical factors in the incidence of such accidents. Therefore, the port equipment and infrastructure must be paid a specific attention with regard to their availability, quantity, maintenance, updating and calibration. The third most critical factor in this regard is affixed to be the human. Which implies that the port authorities shall specifically emphasize on the qualification, experience, training, awareness and responsibility realization of all the staff in general and that associated with the dealings of hazardous cargos specifically. Devising and implementing a port safety management and risk mitigation plan for the hazardous cargos around these critical factors will certainly evade the occurrence of dangerous goods accidents and diminish the severity of consequences.

The results of this study holds practical vitality and are in accordance with the trends reported by other studies observed in the causation factors. The governance and management of the hazardous cargo holds critical prominence in ensuring safety. A study aimed at the port hazardous cargo logistics has reported the governance and management as on the key element of their three-dimensional risk management plan [9]. Likewise, another study aimed at the hazardous cargo handling in a port have reported a positive association between the proper management and hazardous cargo accident risk. They have further reported that proper documentation, its understanding and implementation of a viable management policy can play a significant role in the hazardous cargo accident risk reduction [8]. The following of rules for everyone involved in the hazardous cargo operation and the implementation of properly devised rules and regulations are key factors in hazardous cargo safety at ports.

Similarly, the availability and proper functioning of the related equipment and machinery is also being reported as one of the dominant accident causation factors [8–10]. Likewise, another study aimed at the analysis of hazardous cargo accident causation factors have reported that around 20% of the accidents in their study were associated with equipment, 41% were found associated with improper or illegal commands and operations, while 30% were reported to be associated with inadequate safety management [69]. Similarly, human factor has also been reported as one of the leading accident causation factors. Human error or factor is considered to be one of the highest accident instigation factors in the maritime transportation sector, while in the hazardous cargo accidents it has also been found to be one of the most significant contributing factor [8]. Its role in hazardous cargo accidents was found counting for around 28% of the accidents under investigation and in another study was being considered among the three key components of the hazardous cargo risk management plan [9, 10].

## 5. Conclusion

Safety and environment protection are the most prominent concerns when it comes to the dealing of hazardous cargo in the maritime transportation. In a port environment, the hazardous cargo accident and pollution risk is instigated by different factors in a vaguely manner. This study is aimed at the environment and pollution accident’s risk assessment in a port environment. The methodology adopted is the combination of past accidents and expert judgment for the identification of factors and developing the interdependency connections, while, the model development and inference analysis is done through Bayesian Networks using BayesiaLab software. Past accident reports from 1990 till 2018 were incorporated into a set of variables and the developed model was run in the BayesiaLab environment. The results indicate that without setting evidence at any variable, the probability of an environment accident with considerable damages is 59.80. While, the most prominent contributing factors in this scenario are the human and management with incidence probabilities of 76.8 and 76 respectively. The attitude of the employees dealing with the hazardous cargo along the development and implementation of stringent safety protocols are the most prominent sub factors. If evidence is set at the hazardous cargo environment and pollution accident to occur, the highest change in the contribution probability occurs for the Management.

For a hazardous cargo accident to occur, the role of emergency handling and planning holds critical prominence. If the emergency protocols are designed in wake of the past experiences, concurrence to the latest technologies and latest rules and procedures defined and adopted across the globe, it can significantly reduce the occurrence of such accidents. Moreover, appropriate, timely, well planned and quick emergency response is believed to significantly reduce and control the calamitous consequences of such hazardous cargo accidents. Additionally, the natural factors also an enhanced role in accident causation in this scenario. Natural factors are multifaceted from the perspective of their impact, they not only act as instigation agents in the form of rains, winds, storms, heavy tides, high water velocity, lightening and tsunamis, but also have role in consequence severity. The high tides and water velocity can result in farther and increased spread of oil spills, chemical leakages, and release of other obnoxious materials. Likewise, high winds and storms can result in spread of aerial releases of chemical and gaseous discharges.

A sensitivity analysis was conducted to reveal the most critical factors that could be focused at in circumventing the hazardous cargo accidents and diminishing their catastrophic consequences. The results reveal that the port authorities and concerned government departments shall pay specific attention to the qualification and training of the employees and focus at enhancing their work focus. Similarly, the devising the most advanced and site oriented safety measures and their strict implementation along supervision can significantly evade the accident occurrence. The appropriate port infrastructure and availability of the modern and state of the art equipment also holds critical prominence in evasion of the accidents.

This study has various limitations. All the variables taken into account in this study were considered as binary variables, which can be improved by considering different states accounting for various levels of the variables severity or probability of contribution. Similarly, by enhancing the number of states in each variable, various aspects of a single variable could be considered. Different states like negligible, low, medium, high and severe can be added to each node for its role in the accident causation which will further impart pragmatic knowledge and comprehension of the level of indulgence of each aspect and factor. Moreover, this study focused at the quantitative assessment of the involved factors, hence, further research can be done on various qualitative aspects of the accident causation factors and associated consequences. Moreover, the environment and pollution risk was considered as a broad spectrum variable without dividing it further depending upon the type and severity. Consequence states like life and property losses and environmental damages can be added. Also, further categorization of each of these consequence states can be done on the basis of range of losses incurred. The environment and pollution risk could further be categorized on the basis of its nature like fire, explosion and leakages. Additionally, the spread and damages done by each of these categories could be quantitatively assessed and associated with specified influence of each instigating factor.

## Supporting information

S1 Data Model data. (CSV)
